# Legacy and Emerging Pollutants and Their Effects Through the Lens of Environmental Management

**DOI:** 10.3390/jox15030089

**Published:** 2025-06-09

**Authors:** Christina Emmanouil, Petros Samaras, Dorothea Kassiteropoulou

**Affiliations:** 1 School of Spatial Planning and Development, Aristotle University of Thessaloniki, 541 24 Thessaloniki, Greece; 2 Department of Food Science and Technology, International Hellenic University, 570 01 Thessaloniki, Greece; samaras@ihu.gr; 3 Department of Environmental Sciences, University of Thessaly, 382 21 Volos, Greece; dkasiter@uth.gr

In the quest for worldwide economic development, the principles of sustainable development that involve social justice and efficient environmental protection are sometimes forgotten. One aspect of this problem includes expansive industrialization and urbanization that introduce both legacy [1,2] and emerging pollutants [3,4] into our environment; these pollutants may threaten ecosystems and human health [5]. Addressing this crisis requires a deep understanding of the nature of these pollutants and the deployment of innovative removal strategies [6]. The available tools include sustainable practices, such as the circular economy, that maximize resource efficiency [7] and minimize waste generation [8]. Nevertheless, these novel circular business models of reuse and recycling can also aggravate the (bio)accumulation of well-known or emerging pollutants within a semi-closed ecosystem [9]; hence, this is a double-edged sword. These risks have become a topic of major concern, as chemical or biological agents are widely and increasingly used in many activities, resulting in their continued release and recirculation into the environment through several different routes. The “sinks” of these routes may include air (particulate matter (PM), volatile organic compounds (VOCs), and industrial emissions) [10,11,12], water and sediment (metals, microplastics, persistent organic pollutants (POPs), pharmaceuticals, personal care products) [13,14,15,16], and soil (metals, POPs, polyfluoroalkyl substances (PFAs)) [17,18,19], among others. Pollutants may exert distinct and profound effects on ecosystems. In terrestrial systems, they may disrupt soil microbiota and reduce soil fertility [20] and plant growth or they may bioaccumulate within higher animals affecting reproductive health and longevity. In aquatic ecosystems, they may interfere with the reproduction and nutrition of many species. A recent study revealed that 80% of marine protected areas are exposed to potentially harmful levels of pollutants [21], impacting food webs and biodiversity [22]. In this context, the present Special Issue explores the landscape of classic (legacy) and emerging pollutants, focusing on novel monitoring methods, as well as on less well-studied pollution impacts on organisms and ecosystems. Understanding pollutant behavior across environmental compartments, unraveling their detailed effects on ecosystems, and mitigating their impacts on life will be critical in safeguarding our planet’s future.

In this regard, Campani et al. (Contribution 1) studied fungicide mixtures with multiple active ingredients that may affect bees differently than single active compounds in vineyard agroecosystems. This toxicological research used a multi-biomarker approach and revealed disruptions impairing vital bee functions. The limited available data on the sublethal effects of fungicides on bees are highlighted, requiring more comprehensive evaluation of these chemicals on pollinator health.

In similar terrestrial ecosystems, the exposure of earthworms (*Eisenia fetida*) to short-chain perfluoropropylene oxide acids resulted in significant biochemical and behavioral alterations, even at sub-micromolar concentrations, as shown in the study of Rotondo et al. (Contribution 2). The substance HFPO-DA seems to cause the most pronounced effects, representing a significant environmental contaminant. The ecological risks posed by both legacy and emerging PFAS are crucial, and further research into the long-term impacts on soil ecosystems is required.

In terms of freshwater ecosystems, the buildup of fecal contaminants, due to direct discharge of effluents from poultry farms, was verified in Silway River, Philippines, using advanced microbial quantification protocols (Contribution 3). Simple water parameters (pH, T and TSS level) can create favorable conditions for chicken fecal coliforms to thrive. These findings highlight the ecological risks posed by poultry waste and the need for improved waste management practices.

In terms of marine ecosystems, the exposure of the marine copepod *Tigriopus fulvus* to nine rare earth metals (REEs) demonstrated concentration-dependent increases in mortality and immobilization, with LC50 values ranging from 0.56 to 1.99 mg/L (Contribution 4). The exposure to REEs resulted in a significant slowing of naupliar development. Among the nine REEs, lanthanum proved to be the most toxic, with a high impact on the survival and development of *T. fulvus*, affecting copepod populations.

Burdened marine ecosystems, such as those in the Mediterranean, are highly worthy of investigation, as shown in the study of Kasiotis et al. (Contribution 5), who investigated both organic and inorganic contaminants in fish from the Thermaikos Gulf in Greece. Low concentrations of pharmaceuticals and pesticides were found in *D. labrax* and *S. solea*; however, trace metals were detected more frequently. The risk assessment indicated minimal health risks for adults. Nevertheless, the levels of mercury and arsenic could pose a threat to children. These findings emphasize the need for ongoing monitoring of seafood safety.

Furthermore, juvenile trout was used as a model organism to study the effects of graphene-based nanoparticles (Contribution 6). The exposure resulted in changes to their cardiorespiratory function, swimming behavior, and nesting activity. Graphene oxide enhanced swimming performance in juveniles by as much as 72% while reducing nesting by up to 23%. These alterations indicate potential developmental consequences on both behavior and physiology. Additional studies are required to explore the long-term ecological implications.

Subsequently, another model organism, *Daphnia magna*, was chosen to assess the effects of sublethal exposure to pyriproxyfen over a period of 21 days, in the study of Salesa et al. (Contribution 7). Notable biochemical changes included an increase in LDH activity and a reduction in cholesterol levels at a concentration of 14.02 µg/L, among others. These molecular and physiological changes imply that such biomarkers could act as early warning signals for pyriproxyfen.

Finally, the review paper of Ziegler (Contribution 8) summarizes the progress in toxicity testing utilizing duckweeds (Lemnaceae). Recent research has led to the development of fast protocols, the use of additional species, improved sensitivity, and higher throughput. The incorporation of omics technologies—transcriptomics, proteomics, and metabolomics—paired with FTIR spectroscopy and genotoxicity tests, has broadened the range of biomarker-based evaluations. These advancements allow for a more comprehensive understanding of toxic mechanisms and aid in the detection of unknown contaminants.

On the other hand, assessing environmental behavior rather than toxicity effects, Zhou et al. (Contribution 9) examined microparticle concentrations in the sea–surface microlayer and bulk water of Osaka Bay. Microparticle concentrations were elevated in the surface layer, primarily consisting of smaller particles. The predominant polymer identified was polymethyl methacrylate (PMMA). The findings indicate that antifouling paint from vessels is a significant source of pollution. These results underscore the influence of maritime activities on marine contamination.
